# Variable effects of omaveloxolone (RTA408) on primary fibroblasts with mitochondrial defects

**DOI:** 10.3389/fmolb.2022.890653

**Published:** 2022-08-12

**Authors:** Madleen Zighan, David Arkadir, Liza Douiev, Guy Keller, Chaya Miller, Ann Saada

**Affiliations:** ^1^ Department of Genetics, Hadassah Medical Center, Jerusalem, Israel; ^2^ Faculty of Medicine, Hebrew University of Jerusalem, Jerusalem, Israel; ^3^ Department of Neurology, Hadassah Medical Center, Jerusalem, Israel

**Keywords:** Omavleoxolone RTA408, mitochondria, fibroblasts, ROS, mitochondrial disease, Parkinson’s disease

## Abstract

Omaveloxolone (RTA408) is a second-generation oleanane triterpenoid Nrf2 inducer with antioxidant and anti-inflammatory properties and was reported to improve mitochondrial bioenergetics. It is currently being tested in medical trials for Friedrich ataxia, a genetic, multi-organ disease involving mitochondrial dysfunction. Thus, omaveloxolone could potentially be beneficial for additional disorders involving mitochondrial dysfunction. To this end, we investigated its effect on primary fibroblasts derived from patients with mitochondrial complex I deficiency, mitochondrial cytochrome oxidase deficiency, and two recessive forms of Parkinson’s disease. Patients and control cells were incubated in the presence or absence of 50 nM omaveloxolone for 72 h prior to measurements. Generally, growth on galactose medium and ATP production were unaltered. Mitochondrial membrane potential was slightly but significantly decreased, while reactive oxygen species (ROS) production was variably decreased. Mitochondrial mass and mitochondrial DNA (mtDNA) contents were significantly increased in the patient’s cells. These results were partially confirmed by the results of oxygen consumption studies which disclosed increased maximal oxygen consumption rates in most cells and increased energy status in all treated cells. Further investigation is required to explore the precise effect of omaveloxolone on mitochondrial function in disease.

## 1 Introduction

Omaveloxolone (RTA408) is a second-generation synthetic derivative from oleanolic acid, a natural triterpenoid found in medicinal plants. Derivatives of oleane exhibit anti-inflammatory and anticancer activities due to their binding to the adapter protein Kelch-like ECH-associated protein 1 (Keap1) Keap1 and thereby attenuating the transcription factor, erythroid 2-like 2 (Nrf2) degradation by ubiquitination. Subsequently, the accumulation of factor Nrf2 in the nucleus activates the expression of antioxidant genes, decreases the expression of pro-inflammatory genes, and promotes mitochondrial biogenesis (Uittenbogaard and Chiaramello, 2014; Probst et al., 2015) Omaveloxolone was previously evaluated in humans with advanced solid tumors and was shown, in humans, to be tolerable, supporting further investigation of this compound in cancer therapy (Creelan et al., 2017). Arbeti et al. showed that triggering the Nrf2 antioxidant pathway activators was favorable due to the prevention of lipid peroxidation in cellular mouse models of Friedreich’s ataxia (FA). FA is a common inherited recessive ataxia caused by the mutated *FXN* gene, involving mitochondrial iron accumulation and increased oxidative stress. Subsequently, the same group showed that omavleoxolone had a similar positive effect and also restored mitochondrial respiratory chain complex I (C1) activity, and mitochondrial function, in mouse cells as well as in human FA patient-derived fibroblasts (Abeti et al., 2015; Abeti et al., 2018). Safety, pharmacodynamics, and potential benefit of omaveloxolone were evaluated in two clinical trials in FA patients showing significantly improved neurological function compared to placebo and was generally safe and well-tolerated. It was concluded that omaveloxolone represents a potential therapeutic agent in FA (Lynch et al., 2018; Lynch et al., 2021).

Based on the favorable results, demonstrated by others (Uittenbogaard and Chiaramello, 2014; Abeti et al., 2015; Probst et al., 2015; Creelan et al., 2017; Abeti et al., 2018; Lynch et al., 2018; Lynch et al., 2021), we set out to investigate the effect of omaveloxolone in primary fibroblasts derived from patients with other medical conditions involving mitochondrial dysfunction. Included in this study were fibroblasts derived from patients with two primary, mitochondrial diseases and two patients with genetic forms of Parkinson’s disease (PD) as follows; CI deficiency with fatal encephalomyopathy due to mutated *NDUFAF4* (*C6ORF66*) (Saada et al., 2008), a CI assembly factor; and a relatively mild variant of cytochrome *c* oxidase (COX, mitochondrial respiratory chain complex IV) deficiency due to a mutated *COX4I1*, the common isoform of COX subunit 4*,* which presents with Fanconi anemia-like symptoms (Abu-Libdeh et al., 2017). The two patients with PD were as follows; a juvenile form of PD caused by a biallelic copy-number neutral intragenic inversion in the *PRKN* gene, encoding the E3 ubiquitin ligase Parkin (Mor-Shaked et al., 2021) and a juvenile PD patient due to biallelic mutations in the *PARK7* gene encoding the DJ-1 protein (Namnah et al., 2020) The fibroblasts from these patients were incubated 72 h with or without omaveloxolone and subsequently evaluated for mitochondrial function. The effect on various parameters was variable, but a significant increase in mitochondrial content and mtDNA was noted without a concomitant rise in reactive oxygen species (ROS).

## 2 Materials and methods

### 2.1 Materials

Omaveloxolone (RTA408) (BioTAG, Kfar Yona, Israel) was kept frozen as 10 mM stock solutions in dimethylsulfoxide (DMSO). Mitotracker green (MTG) was obtained from (Molecular Probes, Eugene, Oregon, United States). Tetramethylrhodamine ethyl ester (TMRE) and 2′,7′- dichlorodihydrofluorescein (DCF) were obtained from (Biotium, Harvard, CA, United States), the ATPlite Luminescence Assay System was purchased from Perkin Elemer (Perkin Elmer Israel Hod Hasharon Israel). Tissue culture media were obtained from (Biological Industries-Supleco, Kibbutz Beit Haemek, Israel). The Seahorse XF Cell Mito Stress Test Kit and medium were purchased from Agilent Technologies (Wokingham United Kingdom). The DNeasy Blood & Tissue Kit was obtained from QIAGEN (Venlo, Netherlands) and Sybrgreen, qPCRBIO SybrGreen Blue Mix Hi-ROX was obtained from PCRBIOSTSTEMS (London, United Kingdom). Other chemicals were purchased from Sigma-Aldrich-Merck (Rehovot, Israel) in the purest form available.

### 2.2 Methods

#### 2.2.1 Cell cultures and experimental conditions

Previously established primary fibroblasts cell lines (the study was approved by the local institutional review board and all participants signed informed consent forms before beginning the study (0485-09-HMO, 0393–17-HMO) were maintained in permissive glucose-containing (GLU) DMEM medium containing 4.5 g/L D-glucose and supplemented with 15% fetal calf serum (FCS), 50 μg/ml uridine, and 110 μg/ml pyruvate at 37°C in 5% CO_2_. The cells were seeded at an equal density of 3000 cells/in microtiter wells for mitochondrial function or 15,000 cells/well for oxygen consumption experiments and 100.000 cells in T25 flasks for DNA extraction. The following day, the medium was removed and the wells were replaced with the permissive GLU or a restrictive, glucose-free medium with 5 mM galactose and 5% (FCS), in the presence of the vehicle (DMSO) or omaveloxolone at a final concentration of 50 nM and incubated for 72 h at 37°C in 5% CO_2_.

#### 2.2.2 Assessment of mitochondrial function in microtiter wells

The assays were essentially performed as we have previously described (Douiev et al., 2016; Yu-Wai-Man et al., 2017) as follows; cell growth in GLU and GAL media was assessed by measuring cellular content by methylene blue (MB) staining of basophilic cellular components after fixation. The dye was extracted and measured at 620 nm. The cells were also counted using membrane utilizing permeable and impermeable DNA-binding dyes and was performed using the MUSE count and viability kit MCH100102 (Merck-Millipore United States) on a Guava Muse Cell Analyzer instrument (Luminex, Austin, TX, United States).

Mitochondrial ATP synthesis was measured in permeabilized cells after incubation with glutamate and malate in the presence of ADP. ATP produced in the GLU medium was analyzed by luciferin–luciferase using the ATPlite Luminescence Assay System and presented as relative luminescence units, (RLU) normalized to MB. Reactive oxygen species (ROS) production in GLU medium was determined with DCF fluorimetry at λex 485 nm and λem 520 nm and presented as relative fluorescence units (RFUs) normalized to MB. Mitochondrial content and membrane potential (MMT) in GLU medium were estimated by co-staining with the fluorescent dyes MTG (independent on MMT) on and TMRE (dependent on MMT), respectively, and presented as RFU ratios or normalized to MB. Fluorescence was measured at λex 485 nm and λem 535 nm for MTG and λex 485 nm and λem 590 nm for TMRE. All measurements were performed on a Tecan Spark microplate reader (Tecan Group Ltd. Männedorf, Switzerland).

#### 2.2.3 Mitochondrial content

DNA was isolated from the fibroblasts grown in GLU medium with the QIAGEN DNeasy Blood & Tissue) kit. MtDNA content was determined by real-time quantitative PCR (qPCR) with SyGreen Blue Mix Hi-ROX, using exactly the same primers and conditions described by primers which were specific for the mitochondrial t-RNA leucine gene (*MT-TL1*) and the normalization primers which were specific for the nuclear β-2-microglobulin (*β2M*) gene Venegas et al. (2011). The reaction and analysis were performed on a Step OnePlus RealTime-PCR system (ThermoFisher Scientific, Waltham, MA, United States). The relative mtDNA content was calculated using the delta-delta Ct method formula comparing patients to control cells and treated to untreated cells.

#### 2.2.4 Mitochondrial oxygen consumption and extracellular acidification

Oxygen consumption rates (OCR) and extracellular acidification rates (ECAR) were measured by the Agilent Seahorse XF Cell Mito Stress Test according to the manufacturer’s instructions and as we have previously described (Yu-Wai-Man et al., 2017), using the XF Extracellular 96-well Flux Analyzer (Agilent Technologies, Wokingham, United Kingdom). Briefly, fibroblasts were seeded in quadruplicates on XF96-well plates in GLU medium, in the absence or presence of omaveloxolone for 72 h as described above. Prior to the measurement, the medium was changed to Seahorse XF unbuffered DMEM medium supplemented with 1 mM pyruvate, 2 mM glutamine, and 10 mM glucose, and the plate was equilibrated at 37°C for 1 h. Initially, basal OCR and ECAR were measured, and thereafter oligomycin was injected to a final concentration of 2.5 µM. Subsequently, carbonyl cyanide-4 trifluoromethoxy phenylhydrazone (FCCP) was injected to a final concentration of 2 μM, and maximal oxygen OCR was measured. Finally, 0.5 μM of each rotenone and antimycin were injected to measure non-mitochondrial OCR. After measurements, the wells were stained with methylene blue as described above, and the calculation of all parameters included normalization to cell content. Basal and maximal OCR was calculated after subtracting non-mitochondrial OCR. ATP synthase-dependent OCR was estimated by subtracting basal OCR from OCR in the presence of oligomycin. Spare capacity was calculated by subtracting maximal OCR from basal OCR. The respiratory control ratio was estimated as the maximal/basal OCR ratio. The energy status was visualized by plotting OCR versus ECAR.

#### 2.2.5 Statistical analysis

Experiments were performed in triplicates on two or more independent occasions and presented as mean ± SD. Statistical significance *p* < 0.05 was calculated by unpaired, two-tailed Students’ t-test assuming equal variance using IBM-SPSS™ v.20 software (IBM Corp. Armonk, NY).

## 3 Results

We investigated the effect of omevaloxolone on mitochondrial parameters in order to assess the preliminary therapeutic potential of this compound in diseases involving mitochondrial dysfunction. In this study, we included a variety of primary fibroblasts representing a severe, fatal mitochondrial disease C1 (Saada et al., 2008), a milder mitochondrial disease affecting COX (Abu-Libdeh et al., 2017), and two recessive forms of PD, a common neurodegenerative disease, which has previously been linked to mitochondrial dysfunction and oxidative stress [reviewed in (Zambrano et al., 2022)]. In this preliminary study, we incubated the cells with omaveloxolone in a concentration that was previously reported for the *in vitro* FA studies (Abeti et al., 2015; Abeti et al., 2018) and examined six different parameters to assess different aspects of its effect on mitochondrial function.

First, we examined growth in the GLU medium which was only slightly but not significantly affected as measured by methylene blue (MB) or by viable cell count using the MUSE cell analyzer. Only COX showed a mild 15%–20% decrease (Supplementary Figure S1). As there was no significant difference between MB and viable count, we opted to continue with MB as this method is more convenient to perform in microtiter wells. Additionally, growth was not significantly different, and we did not detect any increased cell death in cells grown up to 15 days (medium change every 3 days) in the presence of omevaloxolone relative to 72 h (results not shown). As our previous experience (Golubitzky et al., 2011) showed that 72 h is the optimal time point to examine growth on GAL versus GLU, the following experiment was performed at this time point.

**FIGURE 1:**
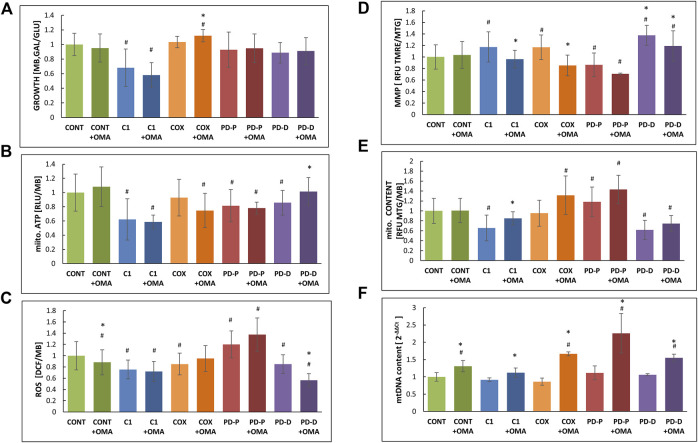
Effect of omaveloxolone on cell growth, ATP production, ROS production, MMP, and mitochondrial and mtDNA content. An equal amount of control (CONT) cells with mitochondrial CI (C1) or COX (COX) deficiency or PD with mutated *PRKN* (PD-P) or mutated DJ-1 (PD-D) was seeded in microtiter wells, cultivated in the presence or absence of 50 nM omaveloxolone (OMA) for 72 h in permissive (GLU) or restrictive (GAL) medium where after the following parameters were measured; **(A)** growth ratio GAL vs. GLU measured by methylene blue staining (MB); **(B)** mitochondrial ATP production measured by luciferin–luciferase (RLU) normalized to MB; **(C)** ROS production measured by DCF and normalized to MB; **(D)** mitochondrial membrane potential (MMP) was calculated with the ratio of TMRE to MTG relative fluorescence units (RFU); **(E)** mitochondrial content was measured by MTG, RFU and normalized to MB. **(F)** mtDNA content was measured by qPCR of the mitochondrial *MT-TL1*gene and normalized to the nuclear *β2M.* Results are presented as mean ± SD relative to controls (*n* = 3). #*p* < 0.05 compared to untreated control mean, *<0.05 compared to the individual cell without additive.

Next, we measured growth in glucose-free medium (GAL), a medium in which cells with impaired oxidative phosphorylation (OXPHOS) are restricted, relative to glucose-containing medium (GLU) where the energy needs are met by glycolysis (Figure 1A). Cell growth on GAL relative to GLU shows markedly impaired growth only in the CI deficient cells, while the growth of PD cells was only slightly but not significantly restricted. Omaveloxolone had no significant effect with the exception of COX where the GAL/GLU ratio was minimally elevated.

We also measured mitochondrial ATP production in permeabilized cells, where CI and PD cells showed decreased baseline ATP production (Figure 1B). Oxamevalone did not significantly alter ATP production, with the exception for PD cells carrying the *DJ1* mutations where ATP production was slightly (15%) but significantly increased. In the COX cells, ATP production showed a decreasing trend, but this was not statistically significant.

Unexpectedly, omaveloxolone decreased ROS only in the controls and DJ-1 mutant cells, while other cells were not significantly affected (Figure 1C). Notably, only *PRKN* mutated cells disclosed an *a priori* elevated ROS level, which was not affected by omaveloxolone.

Mitochondrial membrane potential (MMP) was estimated by calculating the ratio of TMRE which is a mitochondrial marker, dependent on MMP with MGT staining, a mitochondrial dye, independent of MMP as a marker for mitochondrial mass. Unexpectedly, omaveloxalone significantly decreased MMP in most patient cells (Figure 1D) The decreased TMRE: MTG ratio with the largely unaltered ATP production led us to speculate if our observations could be a result of variable mitochondrial content. Accordingly, we calculated mitochondrial content relative to cell mass (Figure 1E), and we also measured mitochondrial DNA (mtDNA) content by real-time quantitative PCR (Figure 1F). Omaveloxolone significantly elevated mitochondrial mass, up to control values in CI cells which had *a priori* decreased mitochondrial content. There was also an increasing but not significant trend in the patient cells including PD-P cells which started out with a higher baseline (Figure 1E). mtDNA content was significantly elevated in all cells including the controls (Figure 1F). Taken together, the results show that omaveloxolone had a significant effect on all cells by elevating mitochondrial content or mtDNA content or both. Interestingly, increased mitochondrial biogenesis is predicted to enhance ROS production, but this was not the case with omaveloxolone.

**FIGURE 2:**
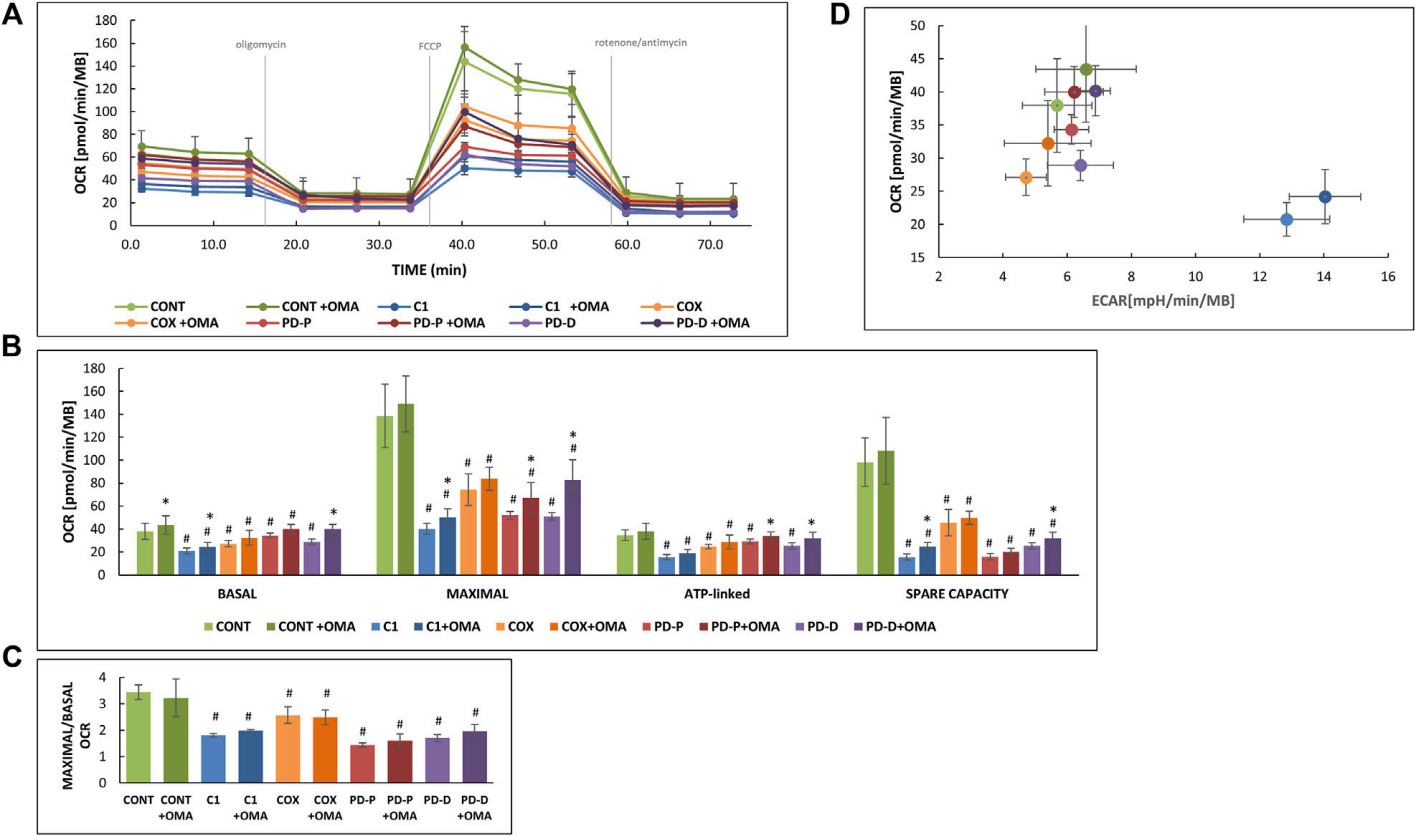
Effect of omaveloxolone on respiration. An equal amount of control cells (CONT) with mitochondrial CI (C1) or COX (COX) deficiency or PD with mutated *PRKN* (PD-P) or mutated DJ-1 (PD-D) was seeded on XF96-well, cultivated in the presence or absence of 50 nM omaveloxolone (OMA) for 72 h GLU medium subsequently oxygen consumption (OCR) and extracellular acidification rates (ECAR) was measured in glucose-containing XF seahorse medium in an XF Extracellular 96-well Flux Analyzer and normalized to cell content measured by methylene blue (MB). **(A)** Depicts the OCR track with time. **(B)** Calculated basal, maximal, ATP-linked OCR and spare capacities. **(C)** Calculated respiratory control ratios (MAXIMAL/BASAL OCR). Results are presented as mean ± SD relative to controls (*n* = 2). #*p* < 0.05 compared to untreated control mean, *<0.05 compared to the individual cell without additive. **(D)** An energy map was constructed by plotting basal OCR representing mitochondrial respiration versus ECAR (before the addition of oligomycin) representing glycolysis. Values toward the upper right quadrant represent more energetic cells.

To complete our studies, we also measured OCAR and ECAR in naïve cells and in cells treated with omaveloxalone (Figure 2). The whole procedure of a typical experiment is depicted in Figure 2A, and calculated values depicted in Figure 2B show significantly decreased basal, maximal, and ATP-linked OCR in the patient’s cell. The severely decreased spare capacity in the patient cells indicates that these cells *a priori* respire near their maximal capacity even under basal conditions. Also, in this system, omaveloxolone showed variable effects. Basal OCR was increased only in controls and in CI cells, while maximal capacity was affected in both CI and PD cells.

Omaveloxolone positively affected ATP-linked OCR in both PD cells, in accordance with previous ATP production experiments (Figure 1B). Spare capacity was slightly but significantly increased only in CI and PD cells with the DJ1 mutations. For reasons we currently are unable to explain, respiratory control (Figure 2C) was decreased in all cells but not affected by omaveloxolone, in discordance with the MMP results (Figure 1D). Nevertheless, the general bioenergetic status of all the cells was improved as depicted in Figure 2D showing elevated basal OCR concomitant with elevated ECAR. This is exemplified by the CI cells; omaveloxolone induced a shift to the right meaning increased acid production as a result of glycolysis and also shifted upwards toward increasing mitochondrial oxygen consumption, meaning that these cells are more energetic.

To summarize, our results show variable effects of omaveloxolone, still with some positive aspects, namely, the effect on the bioenergetic status and the maintained or decreased ROS production despite increased mitochondrial content. PD cells with the DJ1 mutations clearly benefitted the most from omaveloxolone treatment with increased ATP production and ATP-linked OCR with decreased ROS production.

## 4 Discussion

Despite advances in the diagnosis and understanding of the pathomechanisms of diseases involving mitochondrial dysfunction, only a few are responsive to treatment. This is especially true for primary mitochondrial disorders, a common group of inborn errors of metabolism where treatments are mostly supportive and with relatively few ongoing clinical trials. Promisingly, over the past decade, some advances in therapy development mainly with many small molecules are now transitioning from preclinical to early phase human interventional studies, as is omaveloxolone for FA. However, the conclusion of these studies is not yet finalized (Lynch et al., 2018; Pitceathly et al., 2021).

Our results in the CI-deficient cells with severe mitochondrial dysfunction show slightly beneficial effects of omaveloxolone in terms of oxygen consumption and elevated mitochondrial content. Still, there was no significant increase observed neither in ATP production nor in ATP-dependent oxygen consumption. The same is also true for cells derived from the COX patient, which were *a priori* less affected. Thus, the question remains open if elevating mitochondrial content alone could have some beneficial metabolic effect other than increasing ATP production. Indeed, increasing mitochondrial biogenesis has previously been suggested as a therapeutic target in neurodevelopmental disorders and neurodegenerative diseases (Uittenbogaard and Chiaramello, 2014). We preferred DCF for measuring ROS production rather than mitochondria- specific dyes, in order to estimate overall cellular oxidative stress. Notably the two cells with primary mitochondrial diseases did not express elevated ROS. This is in accordance with our previous experience, showing that not all mitochondrial disease fibroblasts express elevated ROS, depending on the nature of the mutation and the antioxidant capacity of the individual cell (Douiev et al., 2016; Yu-Wai-Man et al., 2017). Interestingly, in this study, ROS production levels remained stable or decreased even though mitochondrial content was increased by omaveloxolone.

With respect to the PD cells, there was a notable difference between the two. It seems clear that the PD-D cells harboring *DJ1* mutations gained significant benefit from omaveloxolone with increased ATP production and ATP-dependent OCR concomitantly with decreased ROS. These cells were *a priori* less affected than the PD cells harboring the *PRKN* inversion, which disclosed elevated ROS which was not ameliorated with omaveloxolone. On the contrary, the mitochondrial content and mtDNA were markedly increased in PD-P, Parkin cells. Apparently, this elevation could be responsible for the slight, but not statistically significant, increase in ROS. In these cells, we also noted a discrepancy between mitochondrial ATP production in microtiter wells, which was not affected by omaveloxolone and ATP-linked OCD which showed a slight but significant increase.

Taken together, the only parameter which was common to all cells was the increase in mitochondrial mass and/or mtDNA, which is consistent with the promotion of mitochondrial biogenesis by NRF2 activators (Uittenbogaard and Chiaramello, 2014). Other parameters varied between the cells, and as of now, we are not sure whether these differences stem from the nature of the mutation or from the individual genetic background. This will be addressed in the future, depending on the availability of fibroblasts with the same mutation but with a different genetic background. Our previous study examining fibroblasts with the same homoplasmic mtDNA mutation from different, unrelated individuals suggests that the genetic background does play a significant role (Yu-Wai-Man et al., 2017). Another limiting factor, in studying primary fibroblasts, is that the phenotype might alter with passages and the number of replications, and thus the number of cells is limited. For these reasons, we performed our study with cells from passages 4–6. This also limited our ability to perform more in-depth investigation, including nuclear extraction for examining Nrf2, biochemical, and omics studies. Still from our previous experience, primary fibroblasts maintain the phenotype more consistently than immortalized cells (Saada, 2014). Despite the limitation of this study, it is in line with the suggested Nrf2 signaling pathway as one of the targetable, potential therapeutic pathways in mitochondrial and neurodegenerative diseases (Millichap et al., 2021; Saha et al., 2022).

We conclude that omaveloxolone seems to have a therapeutic potential in certain diseases involving mitochondrial dysfunction and including PD; however, the investigation should be individually evaluated and further studies are required to determine the precise mode of action.

## Data Availability

The raw data supporting the conclusions of this article will be made available by the authors, without undue reservation.

## Supplementary material

Supplementary Figure S1 Effect of omaveloxolone on cell growth using two different methodologies. An equal amount of control cells (CONT) cells with mitochondrial CI (C1) or COX (COX) deficiency or PD with mutated *PRKN* (PD-P) or mutated DJ-1 (PD-D) was seeded in parallel microtiter wells, cultivated in the presence or absence of 50 nM omaveloxolone (OMA) for 72 h in GLU medium. Subsequently, relative cell growth was measured by **(A)** methylene blue (MB) and by **(B)** viable cell counting using a MUSE cell analyzer. The results are presented as mean ± SD, compared to the individual cell without additive *<0.05.
