# Supplementary figures and images for: Variable effects of omaveloxolone (RTA408) on primary fibroblasts with mitochondrial defects
Source: Front Mol Biosci. 2022 Aug 12;9:890653. doi: 10.3389/fmolb.2022.890653 (PMC9411646; doi:10.3389/fmolb.2022.890653)

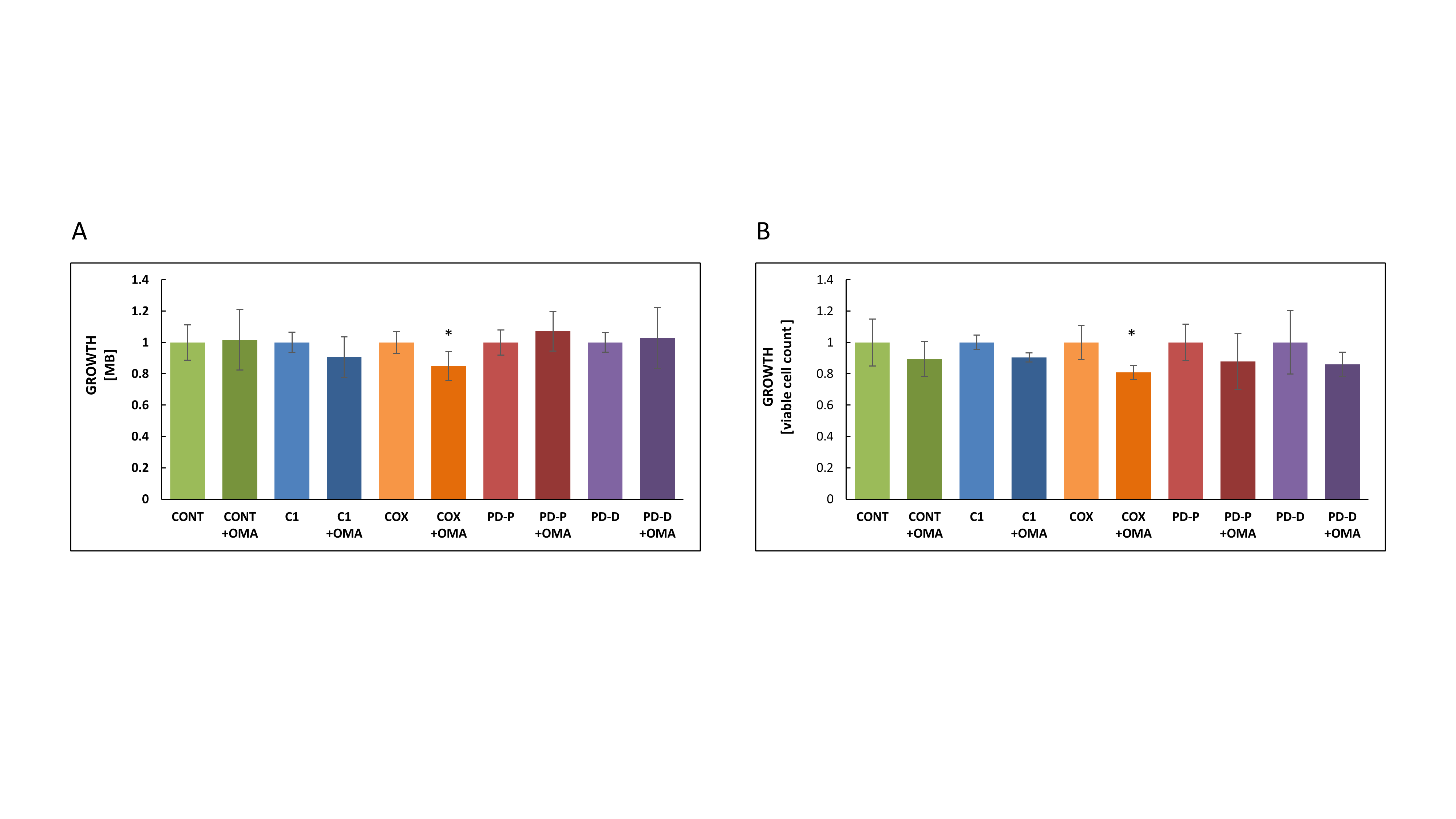

Supplement: Supplementary file 1 [file Image1.TIF]
